# Competency-Based Transitional Support Program for Newly Graduated Nurses: A Program Development Study

**DOI:** 10.1097/jnr.0000000000000697

**Published:** 2025-08-04

**Authors:** Heui-Kyeong KWON, Soohyun KIM, Kyung Yi LEE, Eunhee JUNG, Eunhee LEE

**Affiliations:** 1 Department of Nursing, Seoul National University Hospital, Seoul, Korea; 2 Department of Nursing, College of Nursing, Sungshin Women’s University, Seoul, Korea

**Keywords:** new graduate nurse, transition, competency, residency program, mentoring

## Abstract

**Background:**

Many newly graduated nurses struggle to transition into professional practice due to insufficient training. Nurse residency programs have been implemented in some countries to facilitate smoother role transitions for these nurses. An effective and standardized program must be developed to facilitate the use of support programs by new nurses.

**Purpose:**

The aim of this study was to develop a 1-year competency-based nurse residency program, assess educational needs, and evaluate its effectiveness when applied to newly graduated nurses transitioning into the workforce.

**Methods:**

A competency-based nurse residency program was developed based on the analysis, design, development, implementation, and evaluation of the instructional model. Program outcomes were assessed in accordance with the four-level Kirkpatrick model using standardized posttraining questionnaires, observational surveys, and a review of institutional data. All of the study data were analyzed using descriptive statistics.

**Results:**

The developed 1-year competency-based nurse residency program covered four modules: introductory education, clinical preceptorship, core competency-building education, and mentoring. The outcomes revealed a high satisfaction with introductory education (4.67–4.90 out of 5) among the participants and a significant increase in participant knowledge, skills, and performance. Also, the nurse turnover rate decreased to <10% within 6 months after completing the program. and nursing errors decreased by 1.46 per 100 working days.

**Conclusions:**

A significant percentage of newly graduated nurses leave their jobs during the first several years of work because of difficulties experienced in transitioning into practice. A competency-based nurse residency program that includes emotional support and core competency strength programs was shown in this study to help newly graduated nurses transition smoothly into practice.

## Introduction

Nurse turnover is a significant issue in human resource management and remains unresolved despite the various policies and strategies employed to address it. More than 30% of newly graduated nurses (NGNs) leave their first jobs shortly after being hired, a percentage much higher than the average turnover rate for all nurses (Kovner et al., 2014; Lee, 2019). NGNs experience excessive occupational stress and burnout during their first year of work due to the complexities and uncertainties of fast-paced and unpredictable work environments (Edwards-Maddox, 2022; Fang et al., 2022), which have been identified as key factors influencing turnover (Dall’Ora et al., 2020; Lee, 2019; Ten Hoeve et al., 2020). NGN turnover was reported to be highest in the first year of employment, that is, the period of transition between student and nurse, and declined after that (Lee, 2019; Park & Ko, 2020). Insufficient training has been identified as a critical cause of stress, burnout, and consequent turnover in new nurses during this transitional period (Bakker et al., 2020; Blegen et al., 2017; Lee, 2019).

NGNs are expected to have entry-level competencies upon graduation. However, there are gaps in their role-related knowledge, skills, and clinical judgment. Readiness for practice refers to the ability to safely and competently care for patients by synthesizing theory, skills, attitudes, and values in clinical reasoning in practice settings (Ragsdale & Schuessler, 2021). Higher work readiness facilitates smoother role transition in NGNs. However, most NGNs are concerned they may not have been prepared adequately for the workplace, highlighting the need for strategies to address related issues (Li et al., 2022; Masso et al., 2022). Appropriate education and support for NGNs during their transitional period are critical to building a properly qualified nursing workforce capable of providing high-quality care to patients (Edwards et al., 2015).

Over the past decade, there has been a proliferation of nurse residency programs (NRPs) internationally for NGNs intended to support their transition to practice in the workplace (McCay et al., 2018). Recently, these programs have been applied to specialized nursing activities such as public health, long-term care, and research (Larsen et al., 2018; Legor et al., 2022; Neller et al., 2021). NRPs for NGNs include practical education, mentoring, and emotional support (Chesak et al., 2021; Kramer et al., 2013; McNulty et al., 2022) and are typically applied for periods ranging from 6 months to more than a year. NRPs have also been shown to significantly impact NGN organizational outcomes, including satisfaction, stress, and retention (Asber, 2019; Cline et al., 2017; Van Camp & Chappy, 2017). Despite their positive impact, NRPs are not universally applied in clinical settings in some countries because hospitals lack the appropriate educational staff and budget. Thus, many NGNs still struggle to complete the transition to professional practice due to insufficient training, resulting in excessive workloads, job stress, and burnout (Labrague & De Los Santos, 2020; Ten Hoeve et al., 2020) and leading many NGNs to leave their first jobs.

The complex and multifaceted nature of the transition from student to nurse may limit the widespread implementation of a generalized and formalized NRP. Previous studies have identified wide variations in program components, content, length, number, type of clinical rotations, teaching and learning strategies, and the use of theory in program designs (Anderson et al., 2012; Rush et al., 2019). Although little consensus exists regarding what constitutes best practices for supporting the NGN transition, applying bundled strategies while enhancing the quality of preceptor support has been identified as positively influencing the transitional experience (Rush et al., 2019). However, few studies on the effectiveness of various novel strategies or which specific support strategies are the most effective within the transitional phase have been published. Therefore, this study was designed to develop a 1-year competency-based NRP by analyzing educational needs by period and to evaluate the effectiveness of the developed program in terms of transitioning NGNs successfully into the workforce.

## Methods

### Study Design

A methodological study design and the analysis, design, development, implementation, and evaluation (ADDIE) model (Branch, 2009) were used to develop a competency-based NRP to facilitate the NGN transition process. This study was conducted in three steps: (1) program development (analysis, design, and development), (2) program implementation, and (3) program evaluation.

### Program Development

In line with the ADDIE model, in the analysis phase before designing and developing the program, a focus group interview, two surveys, literature reviews, and outcome evaluations of the pilot version of the NRP were conducted. Information gathered from the focus group interview was used to identify the educational needs of NGNs. Fifteen nurses, including NGNs, preceptor nurses, and nurse managers, participated in the interview. With regard to the two surveys, the first was conducted on 252 NGNs to identify satisfaction with their existing training program and their additional education needs and the second was conducted on 270 NGNs to measure their level of practice readiness. The literature review was conducted to develop the NRP curricular framework. During 2020–2021, a pilot version of the NRP, including standardized introductory education, preceptorship, and mentoring, was implemented with 187 NGNs who were all assigned to intensive care units (ICUs). Because many patients in the ICU are affected by high-severity conditions, ICU nurses must have advanced clinical skills and critical care protocols appropriate to ensure patient safety. Thus, an NRP for ICU nurses was prioritized. The outcomes of the pilot version of the developed NRP were analyzed.

Next, in the design phase, the education team designed the program outline and evaluation method. In terms of program design, the educational team identified the objectives of the NRP and the competencies of NGNs. Subsequently, they designed program modules, training content and strategies, evaluation methods, and teaching methods based on these objectives and competencies. The NRP outcome evaluations were designed using the Kirkpatrick evaluation model (Kirkpatrick & Kirkpatrick, 2016), which has been widely used to evaluate the effectiveness of training programs. This model rates programs based on four levels: reaction, learning, behavior, and results (Kirkpatrick & Kirkpatrick, 2016). Level 1 (reaction) indicates the degree to which participants found the program to be favorable, engaging, and relevant to their jobs. Level 2 (learning) indicates the degree to which the participants acquired the intended knowledge, skills, attitudes, confidence, and commitment based on their participation in the program. Level 3 (behavior) indicates the degree to which participants applied what they learned during the program to their jobs, and Level 4 (results) indicates the degree to which targeted outcomes were achieved as a result of the program (Kirkpatrick & Kirkpatrick, 2016).

After designing the program outline, the education team proceeded to develop the program content and materials based on the results of the abovementioned analysis. A formative evaluation questionnaire and summary evaluation instruments were selected or developed based on program module objectives. To improve the feasibility of use, a panel of five experts, including one nurse educator, one nursing management expert, and three clinical practice experts, was formed to revise the program and verify its validity. The NRP was developed over the first 6 months of 2022 and was revised to improve feasibility for use during the subsequent 2 months afterward.

### Program Implementation

The NRP was implemented on the newly employed NGNs by a tertiary hospital in Seoul, South Korea. All NGNs who joined the hospital after August 2022 participated in the 1-year NRP. A total of 54 NGNs completed the introductory education and preceptorship during the initial phase.

### Program Evaluation

Program outcomes were evaluated in accordance with the Kirkpatrick model. Program satisfaction, that is, reaction (Level 1), was measured using structured questionnaires once each training module was completed. Knowledge and skills (Level 2) were respectively measured by reflection quizzes focusing on the core competencies required of new nurses, implemented and evaluated before and after introductory education, and by an objective structured clinical examination, implemented after the introductory education. Passing and failing grades were determined based on a threshold score of 80. Practical performance, referring to the behavior (Level 3), was self-assessed using the 35-item Nursing Practice Readiness Scale developed by Kim and Shin (2022). This instrument has five subdomains, including clinical judgment and nursing performance (16 items), professional values and attitudes (8 items), patient-oriented attitude (5 items), self-regulation (3 items), and cooperative interpersonal relationship (3 items). Practical performance was assessed using this survey, which was scored on a 4-point Likert scale and demonstrated high internal consistency with a Cronbach’s α of .899.

This survey included both self-assessment and clinical educator evaluation components, with the latter evaluating practical performance using the clinical judgment and nursing performance subdomains of the Nursing Practice Readiness scale (Kim & Shin, 2022). Level 4 (results), including turnover and incidence of nursing errors, was evaluated using hospital data. The turnover rate was calculated as the ratio of resignations out of the 54 NGNs, while nursing error incidence was calculated as the ratio of nursing errors to the average number of workdays for these 54 NGNs. Following the Kirkpatrick model, program outcomes were evaluated at all levels for the 54 NGNs 3 months after NRP implementation.

### Ethical Considerations

Ethical approval for this study was provided by the institutional review board (No. H-2208-099-1350). The education team explained the purpose of the NRP to all of the participants and obtained their informed consent.

### Data Analysis

Data were analyzed using IBM SPSS Statistics 29.0 (IBM Corp., Armonk, NY, USA). Descriptive statistics were used to evaluate the program outcomes, presented as counts, percentages, means, and standard deviations.

## Results

### Educational Needs Assessment in the Analysis Phase

The NGNs, preceptors, and managers participating in the focus group suggested (1) reinforcing initial onboarding education using various teaching methods and (2) standardizing nursing education for medication administration and hospital discharge. The NGNs in the focus group suggested that competency-building be strengthened during the adjustment phase and that emotional support programs should be provided.

Two surveys were conducted in the analysis phase, with 252 NGNs with an average clinical experience of 7.1 months participating in the first. The results of the first survey showed satisfaction with the existing training program to be 3.3 (out of a total possible score of 4). The qualitative data collected from NGNs during the first survey were used to improve the training program. The four most frequently cited suggestions were to (1) improve preceptorship, (2) enhance the specialized nursing education (nursing skills training) provided by clinical nurse educators, (3) customize job-specific training, and (4) extend the advanced clinical training provided by clinical nurse educators. The second survey measured the practice readiness of 277 NGNs whose average clinical experience was 6.7 months. Practice readiness was measured every 3 months and ranged from 82 to 140, with statistically significant differences according to length of clinical experience (*F*=7.11, *p*=.001).

The pilot version of the NRP demonstrated improvement in clinical competency for NGNs in the ICU, although it did not fully address their emotional support needs. Reflecting on these preliminary outcomes, the program was expanded to all hospital units and systematically revised to enhance its overall effectiveness. In addition, recognizing the need for emotional support, a separate psychological support program was introduced to promote socialization, job satisfaction, and organizational commitment.

### Program Outline Based on the Core Competencies for NGNs

An education team and expert panel identified the core competencies of NGNs as (1) nurse professionalism, (2) clinical nursing, (3) interpersonal relationships, (4) problem-solving, (5) scholarship, and (6) leadership (Table 1).

**Table 1:**
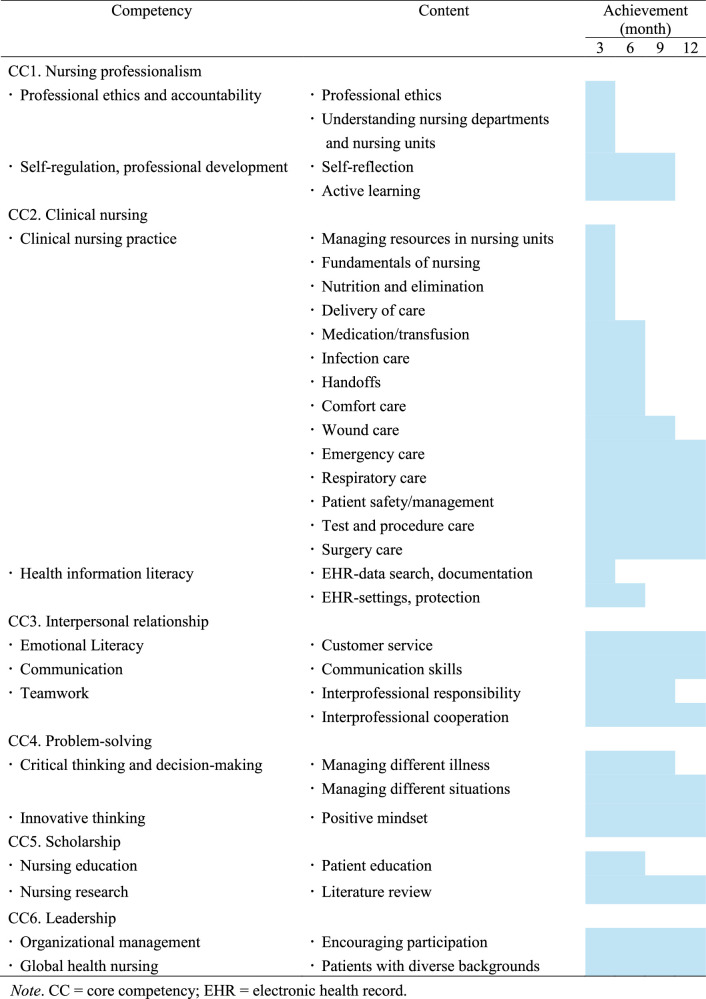
Core Competencies Required of Newly Graduated Nurses

**Figure 1:**
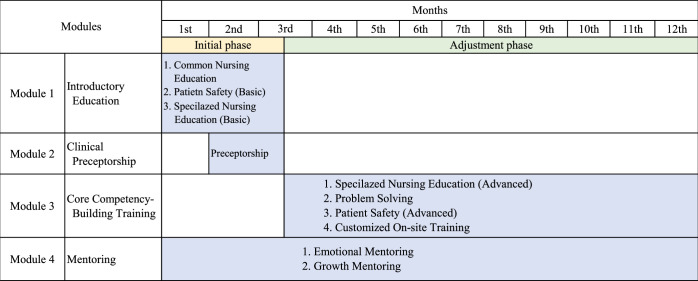
Competency-Based Nursing Residency Program Outline

The program outline was based on the core competencies of NGNs. The NRP period was divided into initial and adjustment phases based on core competencies and achievement time. In addition, NRP modules were designed based on educational needs, such as standardized onboarding education, continuous customized competency-building training, and emotional support programs identified in the analysis phase. To meet these educational needs, three modules were developed in addition to clinical preceptorship, which was part of the existing training program for the NGNs. Thus, the NRP consisted of four modules: (1) introductory education, (2) clinical preceptorship, (3) core competency-building training, and (4) mentoring (Figure 1). The education team provided introductory education for 2½ months just after entering the hospital. After 1 month of introductory education, the NGNs were assigned to a working unit. Depending on the work unit, they were provided preceptorship for 1 or a ½ month. While introductory education and preceptorship were provided within the initial phase, customized competency-building training was provided within the adjustment phase. During the initial phase, NGNs undergo periodic competency evaluations from their preceptors and education team, and the results of these competency evaluations are designed to be used in customized competency-building training. Mentoring was periodically provided throughout the year.

### Program Content

Expert panels and educators developed lesson plans, educational materials, and operational manuals for each module for learners and educators (Table 2).

**Table 2:** Program Content and Educational Methods Per Module in Competency-Based Nursing Residency Program

Module/Program	Core Competency	Subcompetency	Content	Educational Methods
Module 1: Introductory education				
Common nursing education (Basic)	CC1 CC2 CC3	• Professional ethics and accountability • Clinical nursing practice • Health information literacy • Emotional literacy • Communication • Teamwork • Innovative thinking	• Professional ethics • Fundamentals of nursing • Nutrition and elimination • Delivery of care • Medication/transfusion • Infection care • Test and procedure care • Electronic health records • Customer service • Communication skills • Interprofessional responsibility	• Lecture (online/offline) • Discussion • Simulation
Patient safety education (Basic)	CC1 CC2	• Professional ethics and accountability • Clinical nursing practice	• Professional ethics • Medication/transfusion • Patient safety/management	• Lecture (offline) • Case-based learning
Specialized nursing education (Basic)	CC2 CC3 CC4	• Clinical nursing practice • Health information literacy • Critical thinking and decision-making • Communication	• Understanding nursing units • Managing resources • Medication/transfusion • Handoffs • Wound care • Respiratory care • Surgery care	• Lecture • Case-based learning • Simulation • Field training
Common nursing education (Advanced)	CC2 CC3 CC4 CC5	• Clinical nursing practice • Health information literacy • Teamwork • Critical thinking and Decision-making • Organizational management	• Medication/transfusion • Infection care • Comfort care • Emergency care • Respiratory care • Communication skills	• Simulation
Module 2: Clinical preceptorship				
Clinical nursing preceptorship	CC1 CC2 CC3 CC4 CC5 CC6	• Professional ethics and accountability • Clinical nursing practice • Health information literacy • Communication • Critical thinking and decision-making • Nursing education • Teamwork • Organizational management	• Managing resources • Fundamentals of nursing • Delivery of care • Medication/transfusion • Communication skills • Handoff • Managing different illnesses • Patient education • Patients with diverse backgrounds	• One-on-one preceptorship • Bedside teaching • Shadowing and transition competency feedback
Module 3: Core competency-building training				
Specialized nursing education (Advanced)	CC2 CC3 CC4 CC5	• Clinical nursing practice • Health information literacy • Critical thinking and decision-making • Academic	• Respiratory care • Emergency care • Managing different illnesses • Literature review	• Seminar • Peer group study • Simulation
Problem-solving	CC2 CC3 CC4	• Clinical nursing practice • Communication • Teamwork • Critical thinking and decision-making	• Emergency care • Managing different situations • Communication skills • Teamwork	• Case-based learning • Team-based learning
Patient safety (Advanced)	CC1 CC2	• Professional ethics and accountability • Clinical nursing practice	• Patient safety/management • Medication/transfusion	• Case-based learning • Team-based learning
Customized on-site training	CC2 CC3 CC4	• Clinical nursing practice • Communication • Critical thinking and decision-making • Teamwork	• Wound care • Emergency care • Respiratory care • Communication skills • Managing different illnesses • Patient education	• On-the-job training • Shadowing • Competency feedback • Progress report
Module 4: Mentoring				
Emotional mentoring	CC1 CC3	• Self-regulation • Professional development • Communication • Teamwork	• Mind education • Positive mindset • Self-regulation • Communication skills • Interprofessional cooperation	• Lecture • Self-report • Group mentoring • 1:1 mentoring
Growth mentoring	CC1 CC6	• Self-regulation • Professional development • Active learning • Organizational management	• Professional ethics • Understanding nursing units • Self-reflection • Encouraging participation	• SWOT analysis • Reflection essay • 1:1 mentoring

*Note.* CC = core competency; SWOT = strengths, weaknesses, opportunities, and threats.

#### Module 1: introductory education

Module 1 was introductory education, comprising common nursing practice education, specialized nursing education for each department, and patient safety education. The common nursing practice education included basic nursing skill guidelines, such as medication administration principles and procedures, infection control, communication, and patient experience education. It was delivered over 3 days through online education, offline education, discussions, and educational simulations. The specialized nursing education for each department was tailored to the characteristics of each department. Introductory education lasted 3 days for operating rooms, 3 days for wards and emergency rooms, and 10 days for intensive care units. Patient safety education included principles of patient safety, precautions, and tests, and was focused on patient safety incident cases by department.

#### Module 2: clinical preceptorship

In Module 2 (clinical preceptorship), preceptors were assigned to NGNs and took responsibility to train NGNs at the bedside according to the educational goals and content of each period. The preceptors provided systematic training to the NGNs for 1½ months, evaluated their competencies using a structured evaluation system, and provided feedback to the NGNs. All competency evaluation results were reported to the educational team to determine the need for additional competency-building training.

#### Module 3: core competency-building training

Module 3 was conducted in the adjustment phase, which included advanced nursing, advanced patient safety, problem-solving, and individual-based on-site training. This training was customized to the specialized needs of each department using case-based learning techniques to improve situational problem-solving skills as well as customized to each individual learner. In the initial phase, customized core competency-building training was provided for the NGNs based on the competency evaluation results received from the preceptor and education team. To raise awareness of patient safety, patient safety education was conducted, during which patient safety accident experiences were shared and reporting procedure education was provided.

#### Module 4: mentoring

In this module, emotional and growth mentoring was used to enhance interpersonal relationships and nursing professionalism competencies. Emotional mentoring included mind education, self-regulation education, and spike mentoring, while mind education focused on recognizing one’s emotions and needs and sharing emotions with one’s peer group. Self-regulation education was conducted to analyze the abilities of each NGN with regard to learning control, motivation control, behavior control, and resilience. Spike mentoring, which provides emotional mentoring using one-on-one mentoring when experiencing difficulties in the early stages of practical transition, was provided upon request. Growth mentoring includes analysis of one’s strengths, weaknesses, opportunities, and threats (SWOT) as well as career mapping. A reflection essay was used for growth mentoring.

### Program Outcome

**Figure 2:**
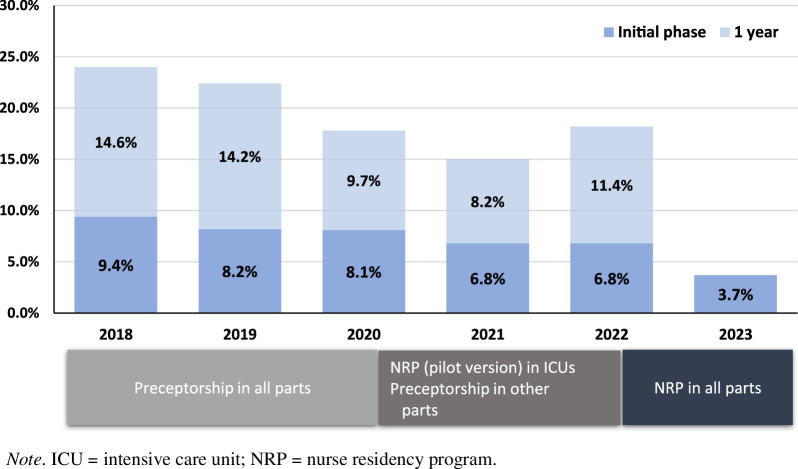
First-Year Turnover Trend for New Nurses

The outcome of the program is presented in Table 3 and Figure 2. Satisfaction with introductory education and preceptorship was measured. Satisfaction with introductory education scored 4.67 out of 5 for common nursing education and 4.90 for specialized nursing education. Satisfaction with preceptorship scored 4.18 out of 5, a lower score than for introductory education. NGNs’ knowledge increased by 52.1% after common nursing education and 40.7% after clinical nursing education. All of the NGNs met the criteria for skill performance after clinical nursing education. As six NGNs resigned during the 3-month study period, practical performance (Level 3) outcomes were measured in 48 NGNs only. The average self-assessment score for practical performance was 102.9 (*SD* = 13.0, min-max: 65–137), and the average practice performance score was 91.8% (min-max: 75%–100%). Lastly, Level 4 results were evaluated during the initial phase. Of the original sample of 54 NGNs, five (9.3%) left their jobs (two [3.7%] in the initial phase). The average career length of the five NGNs who left their jobs was 60.8 days (min-max: 36–92). The 3.7% turnover in the initial stage was lower than in previous years (Figure 2). In the initial phase, the NGNs made a total of seven errors (100% medication errors). In terms of working days, the NGN error rate was 1.46 errors per 100 working days.

**Table 3:** Initial Phase Results for the Participants, August Through December of 2022 (*N* = 54)

Level	Measure	Result
Level 1: Reaction	Satisfaction	• A survey investigated the satisfaction with common nursing education, specialized nursing education, and preceptorship. • Satisfaction scores were 4.67 out of 5 for common nursing education, 4.90 for specialized nursing education, and 4.18 for preceptorship.
Level 2: Learning	1. Knowledge	• Pre-post tests were used to evaluate the knowledge level of new nurses. • Knowledge scores increased to 52.1% after common nursing education and 40.7% after clinical nursing education.
	2. Skill	• Five nursing skills, i.e., patient assessment, handling infusion/syringe pumps, defibrillation, medication administration, and ventilator setup, were assessed through objective structured clinical examinations. • Performance was evaluated according to unique criteria that matched the skill being assessed. If all criteria were performed, it was evaluated as passed. • All new nurses met the passing criteria after clinical nursing education.
Level 3: Behavior	Practical performance	• A practical performance evaluation was conducted for 48 new nurses, which included self-assessments and evaluations by the education team. • The average practice performance score was 91.8% (min-max: 75%–100%). • Self-assessment of practical performance was 102.9 (*SD*=13.0, min-max: 65–137).
Level 4: Results	1. Turnover	• Among 54 new nurses, five (9.3%) left their jobs within 6 months, of which two (3.7%) left in the initial phase. • The average career length of the five nurses who left their jobs was 60.8 days (min-max: 36–92).
	2. Nursing error	• Seven nursing errors occurred within 6 months of employment. • All errors were related to medication errors, including one case of wrong patient (14%), four cases of wrong drugs (57%), and two cases of wrong doses (29%). • The nursing error rate per new nurse was 1.46 per 100 working days.^a^

^a^ Working days were calculated as ~20 days per month, excluding weekends and national holidays.

## Discussion

The competency-based NRP developed in this study achieved several positive outcomes during the 6 months at all levels evaluated, including program satisfaction, knowledge, practice performance, and turnover and incidence of nursing errors, respectively. The 6-month turnover rate was 9.3%, of which 3.7% occurred within the initial phase. As many previous studies have reported NRP to be effective in reducing turnover in the first year (Asber, 2019; Pelletier et al., 2019; Van Camp & Chappy, 2017), the post-NRP turnover rate in this study was also expected to be lower than before NRP implementation. In the year NRP was introduced in the ICU in this hospital, the turnover rate for NGNs was 11.4%, which is significantly lower than the average turnover rate for NGNs in Korea of 18%–25% (Cho et al., 2012; Lee, 2019) and similar to the turnover rates reported by other studies examining the effectiveness of NRPs (e.g., Van Camp & Chappy, 2017). Because the characteristics of NGNs differ year to year and various external factors related to turnover, such as salary level, hospital size, and nurse staffing (Lee, 2019), were not controlled, the decreased turnover rate cannot be attributed solely to the effect of NRP. However, it is noteworthy that turnover in the initial phase decreased after NRP implementation, including the pilot version. In terms of longitudinal trends in the turnover rate of NGNs in this study, the turnover rate, which usually exceeded 20%, fell to 10%, which may be attributed to the systematic management of NGNs for a year in the residency program. With the NRP being extended across all clinical settings and sustained over multiple years, a gradual decline in the turnover rate among newly graduated nurses is anticipated.

Similar to previous studies (Van Camp & Chappy, 2017), this study also found that the turnover rate remained low during the NRP implementation period. However, to clearly evaluate the sustainability of the program’s outcomes, it is necessary to analyze the turnover rate trend over a 2-year period. Several previous studies have reported the turnover rate of NGNs at 2 years (Ackerson & Stiles, 2018; Friday et al., 2015; Varner & Leeds, 2012). However, those studies do not consistently show whether the residency program outcomes persisted for 2 years. Most 1-year programs had a limited impact on 2-year turnover (Ackerson & Stiles, 2018; Anderson et al., 2012; Van Camp & Chappy, 2017). In studies showing improvement or the maintenance of effectiveness for 2 years, the program period was longer than 12 months (Bratt, 2009), or additional programs were provided (Varner & Leeds, 2012). Systematic management, including continuing education, should be continuously provided for nurse retention. Therefore, it is necessary to evaluate the duration of the effect of the NRP developed in this study by monitoring the 2-year turnover rate.

The 1-year NRP showed positive results in patient safety, reducing the incidence of adverse events for NGNs. As no previous studies have reported on the impact of residency programs on patient safety events, this result cannot be compared with other studies. This enhancement in patient safety may be attributable to improvements in patient safety core competencies, such as skills, critical thinking, communication, problem-solving, and decision-making. NRPs have previously been reported to enhance various core competencies required for practice in NGNs (Bolden et al., 2011; Edwards et al., 2015). In particular, the program developed in this study periodically evaluated the practical performance of core competencies using multifaceted evaluations, including self-assessments, preceptor evaluations, and manager evaluations. Subsequently, individualized continuing education tailored to the observed outcomes was provided. This approach of providing individualized training based on periodic and objective evaluations helped develop core competencies in the NGNs, resulting in high-quality and safe nursing care. In addition to core competencies, the NRP in this study also incorporated a coaching and mentoring module, which received high satisfaction scores from participants. Emotional support programs, such as mentoring and mindfulness training, are a key component of NRPs designed to support NGNs as they transition into the workforce. Previous studies reported that emotional support programs significantly improved NGNs’ stress, mindfulness, resilience, and burnout (Chesak et al., 2021; McNulty et al., 2022). During the transitional period, new nurses experience significant physical and emotional stress (Han et al., 2022). Thus, mentoring programs for psychological support are ultimately helpful in promoting a smooth transition into practice (Jangland et al., 2021).

While NRPs are provided in many countries, evidence of standardized programs is lacking. In this study, the ADDIE model was used to develop an NRP. Various groups, including preceptors, nurse managers, and NGNs, analyzed NGN educational needs and core competencies. The data analysis results were used to design four NRP modules: introductory education, preceptor training, core competency-building training, and mentoring. In particular, the developed NRP enhanced education during the adjustment phase, which is a strength of this program compared with traditional transitional-support interventions such as orientation, introductory programs, and preceptorship (Edwards et al., 2015; Rush et al., 2019). During the adjustment phase, individualized continuing education contributed to strengthening the core competencies of NGNs, and mentoring contributed to emotional support and career planning. While most residency programs include only mentoring for emotional support (Edwards et al., 2015), in this study, various aspects of transition, including career coaching and mentoring, were considered. One of the key components of a successful, sustainable NRP is a structured program with defined outcomes to promote clinical competence, safe patient care, and professional development (Chant & Westendorf, 2019). Therefore, this program contains components necessary for a successful, sustainable NRP.

This study developed a transition-supporting program, the NRP, using a systematic analysis of educational needs and core competencies. The most important aspect of this study was that a competency-strengthening program was systematically designed and developed based on the core competencies of NGNs and achievement time. Notably, the NRP developed in this study systematically designed customized competency-building training through periodic competency evaluations conducted during the adjustment phase to ensure that most NGNs achieved the requisite level of competency. The methodology used to develop the systematic, customized competency-building program in this study may be used in various nursing education contexts. The 1-year competency-based NRP developed in this study decreased turnover and safety accidents among NGNs. Transition-supporting programs for NGNs are essential from both organizational and patient safety perspectives. Therefore, hospitals should proactively implement NRPs to foster a well-prepared and competent nursing workforce.

This study had some limitations. Although NRP was found to positively affect NGN turnover for 1–2 years, the actual duration of program effectiveness was not determined. Thus, further studies should analyze longitudinal turnover trends and develop additional programs to sustain the NGN retention effect. As NRPs require significant manpower and funding, it is not certain whether the standardized residency program developed in this study can be extended to hospitals with significantly limited resources. Thus, the program should be modified for various hospital environments to maximize applicability. Lastly, this study focused on the effect of the program. However, the program’s cost-effectiveness must be verified to promote expansion in the future.

### Conclusions

The 1-year competency-based NRP developed in this study was designed systematically based on the core competencies of NGNs and their educational needs. The developed program includes regular core competency-building training and mentoring, as well as strengthening existing training programs such as preceptorship and introductory training. The NRP improved the core competencies of NGNs, leading to reduced turnover and safe nursing care. NGNs have a high rate of turnover during their initial employment period due to difficulties faced in transitioning to practice. The NRP, including emotional support and core competency-strengthening, helps NGNs transition into practice smoothly. Based on its effectiveness, nurse managers and hospitals should develop and implement the NRP using this study’s methodology and program contents to achieve a better transition to practice for NGNs.
